# Case Report: Mammary Paget’s disease with multifocal microinvasive carcinoma and extensive lymph node metastasis: therapeutic challenges and insights from a case of stage pT1mic pN3c cM0

**DOI:** 10.3389/fonc.2025.1727016

**Published:** 2026-01-21

**Authors:** YiFan Luo, ZhiYu Liu, Jing Luo

**Affiliations:** 1 School of Medicine, Southwest Medical University of China, Department of Breast Surgery The Affiliated Hospital of Southwest Medical University, Luzhou, China; 2 Department of Breast Surgery, Sichuan Academy of Medical Sciences & Sichuan Provincial People’s Hospital, Affiliated Hospital of School of Medicine, University of Electronic Science and Technology of China, Chengdu, China

**Keywords:** ductalcarcinoma in situ, HER2-overexpressing breast cancer, mammary Paget’s disease, multifocal microinvasive carcinoma, preoperative imaging limitation, sentinel lymphnode biopsy

## Abstract

**Background:**

Mammary Paget’s Disease (MPD) is a rare subtype of breast cancer, accounting for 1%-4% of all breast cancers. Controversy remains regarding whether sentinel lymph node biopsy (SLNB) is necessary for MPD patients undergoing breast-conserving surgery (BCS) when imaging studies fail to detect deep invasive carcinoma, and this controversy lacks support from specific case evidence.

**Case Summary:**

A patient presented with “recurrent left nipple fissure for 3 years and eczematous changes for 3 months.” Preoperative biopsy at another hospital confirmed MPD; imaging showed no deep mass. Postoperative pathology revealed left breast MPD associated with multifocal microinvasive carcinoma, accompanied by metastases to left axillary lymph nodes (6/8), left subclavian lymph nodes (2/3), and left supraclavicular lymph nodes (1/3). The pathological stage was pT1mic pN3c cM0. No recurrence was observed 6 months after adjuvant therapy with the TCbHP regimen plus capecitabine consolidation therapy.

**Conclusion:**

Although no definite mass was identified on breast magnetic resonance imaging (MRI) in this case, SLNB and subsequent pathology confirmed extensive lymph node metastasis (pN3c). Omission of SLNB could have led to understaging and compromised treatment decision-making. This single case may suggest that SLNB holds significant staging value for MPD patients with no obvious breast mass on imaging. It provides hypothesis-generating, practical evidence for addressing this controversial clinical issue, warranting further investigation in larger cohorts.

## 1 Introduction

Mammary Paget’s disease (MPD) is an extremely rare malignant breast tumor, accounting for only 1%–4% of all breast cancers (1). Its typical clinical manifestations include pruritus, erythema, erosion, or ulceration of the nipple-areola complex (2).

A 38-year-old female patient presented to our institution with the chief complaint of recurrent left nipple fissure for 3 years and eczematous changes over the left nipple-areola complex for 3 months. On physical examination: the left nipple showed retraction; centered on the left nipple, an approximately 3 cm × 3 cm area of eczematous changes was observed on the breast skin; two lymph nodes with a long diameter of ~1 cm were palpable in the left axilla, and these nodes exhibited clear borders, good mobility, hard consistency, no fusion, and no tenderness. No abnormal findings were detected in the right breast or right axilla, and the remainder of the physical examination was unremarkable.

Subsequently, the patient underwent partial nipple skin biopsy at a local hospital. Immunohistochemical (IHC) staining of the biopsy specimen confirmed findings consistent with MPD, with the following molecular profile: estrogen receptor (ER) negative; progesterone receptor (PR) negative; human epidermal growth factor receptor 2 (HER2) positive (3+); and Ki-67 proliferation index of 75%.

Current guidelines suggest that SLNB may be omitted in cases of pure MPD (2). However, MPD is often associated with ductal carcinoma *in situ* (DCIS) or invasive breast cancer (IBC, 3), and preoperative imaging is insensitive for detecting deep lesions, failing to completely rule out the possibility of invasion (4). Therefore, the necessity of SLNB for MPD patients without radiological evidence of breast cancer remains a clinical controversy. The Chinese Expert Consensus on the Diagnosis and Treatment of Mammary Paget’s Disease (2025 version), which references the NCCN Guidelines and DCIS management principles, states that if imaging excludes DCIS or IBC, some experts support omitting SLNB during BCS (2).

This report describes this case of MPD with multifocal microinvasive carcinoma, where breast MRI showed no definite mass but only thickening of the areolar skin and nodular enhancement posterior to the areola. SLNB and subsequent surgery confirmed supraclavicular lymph node metastasis. Through the diagnosis and treatment process of this case, we discuss the clinical value of SLNB in MPD patients with negative imaging findings, provide case evidence for resolving the aforementioned controversy, and enhance clinical awareness of the lymph node metastasis risk of MPD associated with microinvasive carcinoma.

## 2 Case presentation

### 2.1 Symptom on set and progression

A 38-year-old female was admitted to our hospital due to “recurrent left nipple fissure for 3 years and eczematous changes for 3 months.” She first noticed the left nipple fissure accidentally after lactation, accompanied by mild nipple discomfort but no nipple discharge or bleeding. Topical medications provided no significant relief. Subsequent recurrent fissures led to obvious retraction of the left nipple compared with the right. Three months prior to admission, the left nipple fissure recurred with pruritus, and the areola developed erythema, papules, and exudative changes.

### 2.2 Initial pathological diagnosis

The patient underwent partial nipple skin biopsy at a local hospital; Immunohistochemical (IHC) results supported a diagnosis of MPD, with ER-negative, PR-negative, HER2-positive (3+), and a Ki-67 proliferation index of 75%. She was referred to our hospital for further treatment.

### 2.3 Patient history

The patient had no history of breast trauma. Menarche occurred at 14 years of age, with regular menstrual cycles (approximately 28 days). Her last menstrual period at the initial diagnosis was December 30, 2024. There was no family history of malignant tumors.

### 2.4 Physical examination

Physical examination revealed retraction of the left nipple, with eczematous changes involving an approximately 3 cm × 3 cm area of skin centered on the nipple. Two left axillary lymph nodes (maximum diameter ~1 cm) were palpable, with clear borders, good mobility, hard texture, no fusion, and no tenderness. No obvious abnormalities were found in the right breast or right axilla; other physical examination findings were unremarkable.

### 2.5 Color doppler ultrasound

Color Doppler ultrasound showed enlargement and hyperemia of the left nipple (size: ~21 × 11 mm) with visible blood flow signals, but no definite space-occupying lesion in the deep breast tissue. A few lymph node echoes were detected in the left axilla, with the largest measuring ~14 × 7 mm, showing cortical thickening (maximum thickness: ~2 mm) and clear cortex-medulla differentiation. No definite lymph nodes were found in the bilateral neck.

### 2.6 Mammography

**Figure 1:**
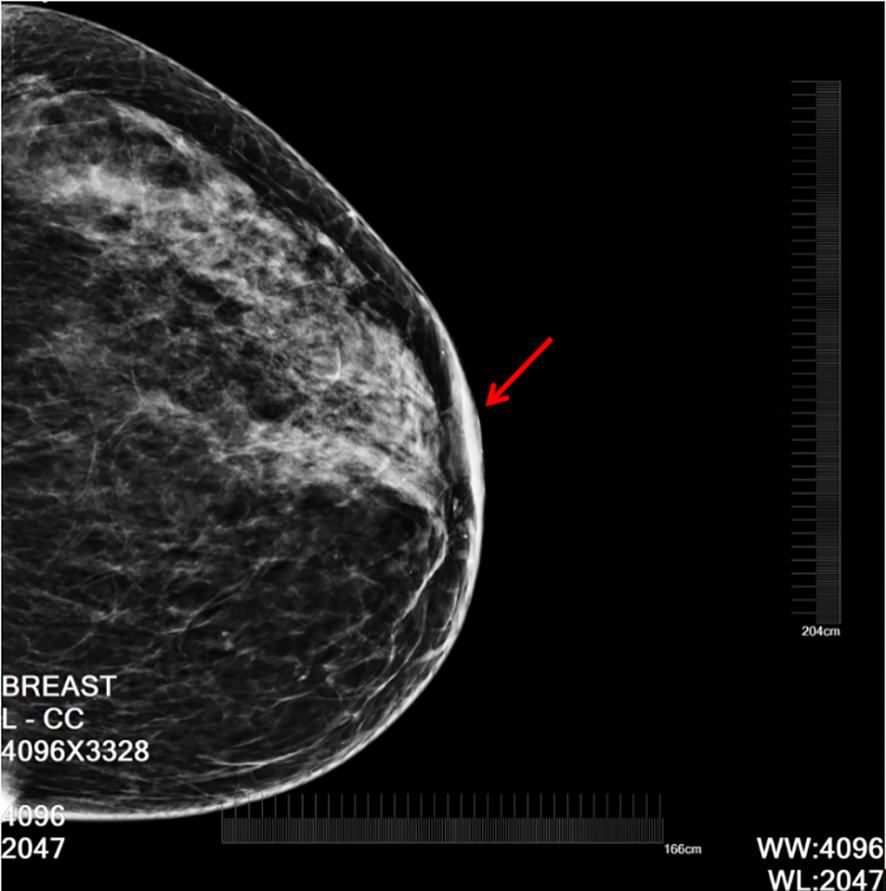
The arrow indicates left nipple retraction with local skin thickening.

Mammography showed that the left nipple was larger than the contralateral nipple, and the areolar and adjacent skin were significantly thicker than the contralateral side. No obvious mass shadow was observed in the left breast (**Figure 1**).

### 2.7 Contrast-enhanced MRI

**Figure 2:**
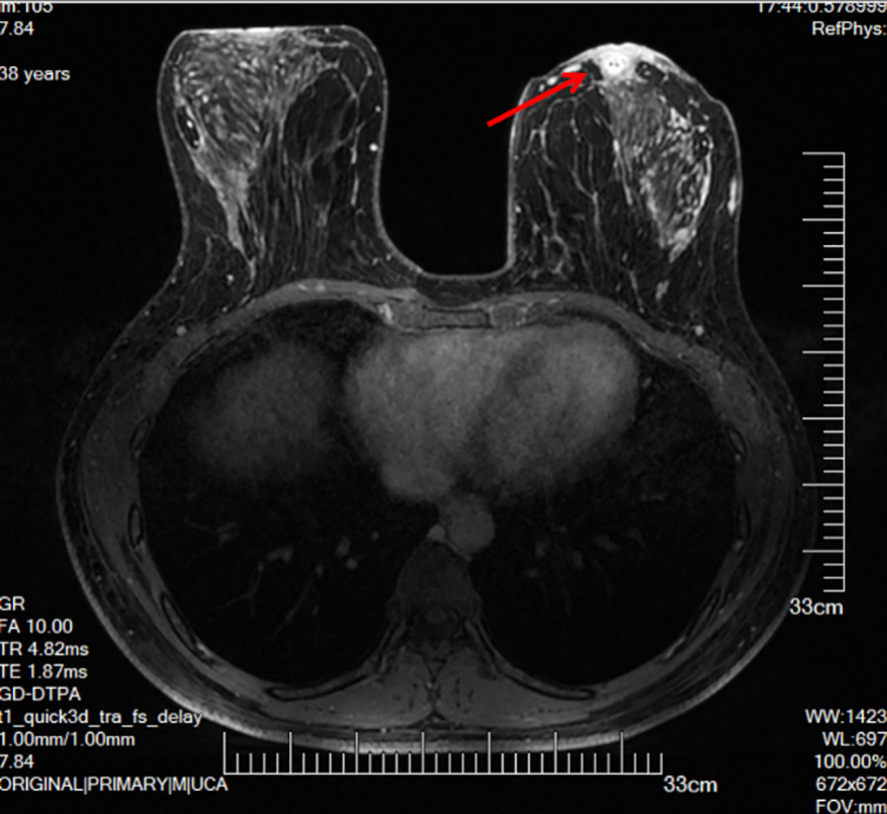
The arrow indicates thickening of the left areolar and surrounding skin with nodular enhancement posterior to the areola.

**Figure 3:**
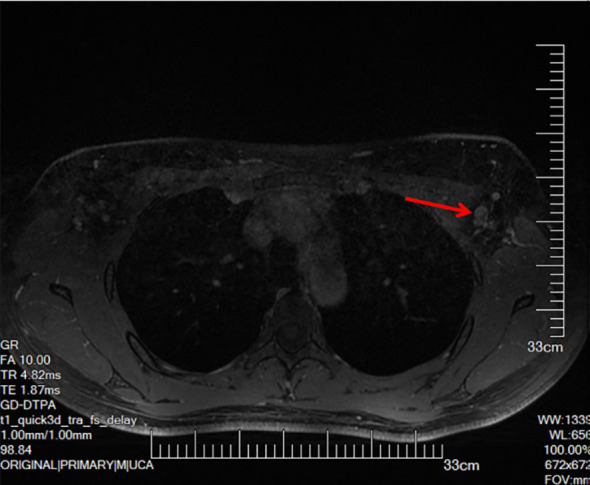
The arrow indicates enlarged lymph nodes.

Contrast-enhanced MRI showed thickening of the left areolar and surrounding skin, with nodular enhancement posterior to the areola, but no obvious mass in the breast (**Figure 2**). Multiple lymph nodes were visualized in the left axilla, with the largest measuring ~10 × 7 mm (**Figure 3**).

### 2.8 Differential diagnosis

The diagnosis of MPD requires careful differentiation from other conditions that can cause nipple-eczematous changes. Key considerations included eczema/dermatitis, contact dermatitis, and nipple adenoma (5). **Eczema or Dermatitis**: Generalized eczema or dermatitis often presents with bilateral, symmetrical involvement of the breasts, characterized by pruritus, erythema, and scaling. Symptoms typically respond to topical corticosteroid therapy. In contrast, this patient had strictly unilateral​ disease with irregular borders and nipple erosion that showed no improvement with topical steroids, which is highly atypical for benign eczema and raised the suspicion of malignancy (5).**Contact Dermatitis**: This condition typically has a clear history of exposure to an irritant or allergen. The rash is usually confined to the contact area and often improves after the offending agent is removed. Our patient had no such exposure history, and the lesions showed persistent progression, making contact dermatitis an unlikely diagnosis (6).**Nipple Adenoma**: This is a benign tumor that usually manifests as a palpable nodule or mass in the nipple. Its surface is generally smooth without erosion, and imaging studies often reveal a well-circumscribed mass. The patient’s presentation, featuring nipple destruction rather than a discrete mass, was inconsistent with the typical features of a nipple adenoma (7).

### 2.9 Diagnostic challenges

#### 2.9.1 Limitations and false-negative potential of preoperative imaging

A key diagnostic challenge was the discrepancy between the negative imaging findings and the high clinical suspicion. Despite comprehensive evaluation with mammography, ultrasound, and MRI, no definite mass was identified. This underscores a critical limitation of current imaging modalities in evaluating MPD. Specifically, contrast-enhanced MRI, while highly sensitive for invasive carcinoma, has limited resolution for detecting microinvasion or extensive DCIS when not forming a discrete mass (8, 9). Microinvasive carcinoma foci (<1 mm) can evade detection due to their minute size and diffuse growth pattern. Furthermore, DCIS often presents as non-mass-like enhancement, which can be subtle and difficult to distinguish from background parenchymal enhancement, especially in patients with dense breast tissue (4, 9). Therefore, a negative MRI cannot reliably exclude the presence of occult invasive disease in the context of MPD (8).

#### 2.9.2 Rationale for surgery

The decision to proceed with SLNB and an extended surgical resection was not based on definitive radiographic evidence of invasion, but on a comprehensive synthesis of high-risk clinical, pathological, and suspicious imaging findings. This aggressive diagnostic approach was deemed necessary to obtain definitive pathological staging and avoid the significant risk of understaging. The rationale was predicated on several key factors:

**Aggressive Tumor Biology**: The initial biopsy revealed an HER2-positive (3+) subtype with a high Ki-67 index (75%). This biological profile is strongly associated with a high propensity for lymphatic invasion and metastasis, independent of the size of the primary lesion (10, 11). Relying solely on imaging to exclude invasion in this context was considered unreliable.

Suspicious Radiologic Findings: Although no discrete mass was identified, contrast-enhanced MRI demonstrated nodular enhancement posterior to the areola. This finding, while not diagnostic, raised a strong suspicion for an underlying high-grade ductal carcinoma *in situ* (DCIS) or even occult invasion, serving as a critical “red flag” that could not be ignored (4, 9).

Clinical High-Risk Features: The patient’s young age (38 years old) and the central/subareolar location of the disease, an area rich in lymphatic drainage, are established risk factors for more aggressive tumor behavior and a higher likelihood of lymph node metastasis (12, 13).

Imperative for Pathological Staging: Given the inherent limitations of preoperative imaging in detecting microinvasion and the constellation of high-risk features, we concluded that histopathological examination of the surgical specimen could provide a definitive diagnosis and accurate staging. SLNB was essential to rule out nodal metastasis, as any lymph node involvement would be a critical determinant for adjuvant therapy. Concurrently, the extended resection ensured complete removal of the nipple-areola complex and underlying tissue, allowing for thorough pathological assessment to identify or rule out any invasive foci.

In summary, the multi-modal surgical strategy (SLNB and extended resection) was driven by the imperative to obtain accurate pathological staging in a patient with high-risk features, despite negative conventional imaging. This approach was crucial for preventing understaging and guiding appropriate, stage-directed adjuvant therapy.

### 2.10 First surgery and pathology report

The patient underwent a left breast partial extended resection (excluding the nipple-areola complex and underlying glandular tissue) without preserving the nipple-areola complex, combined with SLNB. Intraoperative frozen section pathology showed 2 macrometastases and 2 micrometastases among 4 sentinel lymph nodes examined. According to the Guidelines for the Diagnosis and Treatment of Breast Cancer by the Chinese Anti-Cancer Association, left axillary lymph node dissection (ALND) was added intraoperatively.

Final pathology results showed MPD changes in the left nipple and areola, with DCIS involvement in the dermis and multifocal DCIS in the breast tissue. Notably, among 8 axillary lymph nodes dissected, 6 had macrometastases; both of the 2 dissected left subclavian lymph nodes had macrometastases. IHC of the metastatic lymph nodes showed results almost consistent with those of the nipple biopsy specimen.

### 2.11 Postoperative PET-CT

Postoperatively, the patient underwent 18F-FDG PET/CT, which revealed 1) A nodular focus with increased radiotracer uptake in the outer quadrant of the left breast; 2) Multiple lymph nodes visualized in the left supraclavicular fossa, posterior to the clavicle, and deep to the left pectoralis major muscle, with obvious increased radiotracer uptake (most prominent in the left supraclavicular fossa), suggestive of lymph node metastasis; 3) No other tumor signs.

### 2.12 Second surgery and pathology report

Subsequently, the patient underwent a second surgery, which included left total mastectomy, radical left cervical lymph node dissection (left neck Level IV and V), and dissection of lymph nodes between the left pectoralis major and minor muscles. Extensive sampling and histological sectioning of the mastectomy specimen were performed to screen for any invasive lesions. The histopathological examination confirmed the presence of multifocal, high-grade DCIS. Careful evaluation identified multiple, discrete microinvasive carcinoma foci. While an exact count was challenging due to the minute and scattered nature of these foci, at least three distinct foci were confirmed, with the largest individual focus measuring less than 1 mm in its greatest dimension, consistent with the definition of microinvasive carcinoma according to the AJCC 8th edition staging system (14). Critically, no evidence of lymphovascular invasion was identified upon comprehensive histological analysis. IHC results were consistent with those of the previous axillary lymph node examination. One of the 6 dissected left supraclavicular lymph nodes (neck Level V) had macrometastasis. The postoperative pathological diagnosis was pT1mic pN3c cM0 (Stage IIIC, HER2-overexpressing subtype).

### 2.13 Postoperative systemic therapy and rationale

Based on the advanced pathological stage (pT1mic pN3c), HER2-positive subtype, and high nodal tumor burden, the multidisciplinary team (MDT) decided on an intensified, stage-appropriate adjuvant regimen. The rationale for each component of the treatment strategy is outlined below:

Dual Anti-HER2 Therapy Combined with Chemotherapy (TCbHP Regimen): The patient commenced a systemic therapy regimen consisting of nab-paclitaxel (400 mg), carboplatin (700 mg), trastuzumab (480 mg), and pertuzumab (loading dose 840 mg, followed by a maintenance dose of 420 mg). This TCbHP regimen was selected because the presence of lymph node metastases (pN3c) and the HER2-positive status define a high-risk population for which the combination of a taxane and platinum-based chemotherapy with dual HER2 blockade is a standard, evidence-based intensive adjuvant therapy, aiming to maximize the reduction of recurrence risk (15).

Comprehensive Radiotherapy: Beginning in July 2025, the patient underwent adjuvant radiotherapy. The target volumes included the whole breast (post-mastectomy chest wall), supraclavicular, and infraclavicular nodal regions, with an additional boost to the tumor bed. The total radiation course comprised 30 fractions, delivering a cumulative dose of 6000 cGy (200 cGy per fraction). The decision for this comprehensive radiotherapy field was based on the pN3c stage, making irradiation of these nodal basins standardly recommended to achieve optimal regional control (16, 17).

Consolidation Therapy with Capecitabine: From September 2025, the patient received adjuvant consolidation therapy with capecitabine. This decision was grounded in the high nodal tumor burden (pN3c), a strategy supported by evidence for high-risk breast cancer patients, aiming to further mitigate the risk of distant recurrence (18).

Omission of Endocrine Therapy: Given the hormone receptor-negative status (ER-negative, PR-negative) confirmed on repeated pathological examinations, a multidisciplinary team (MDT) assessment concluded that endocrine therapy would offer negligible benefit, and it was therefore omitted (19).

In summary, the treatment strategy was entirely tailored to the patient’s true disease extent, which was definitively established through surgical staging. This case underscores that accurate pathological staging, achieved via SLNB and subsequent procedures, is critical for guiding appropriate, potentially curative, multimodal therapy in patients with MPD and high-risk features.

### 2.14 Follow-up

The patient was followed up at our institution at 1.5-month intervals postoperatively. Surveillance included ultrasonography of the surgical site, complete blood count, and comprehensive metabolic panels. A follow-up FAPI PET/CT scan performed in July 2025 showed no evidence of local recurrence or distant metastasis. The most recent examination in December 2025, via ultrasound, again confirmed no signs of recurrence or metastasis. The most notable adverse effects following chemotherapy were gastrointestinal toxicity and mild myelosuppression, which were effectively managed with symptomatic support including tropisetron and pegylated recombinant human granulocyte colony-stimulating factor. The affected arm shows no signs of lymphedema, with full range of motion and normal function, allowing complete self-sufficiency.

### 2.15 Patient perspective and psychosocial outcomes

The diagnosis of breast cancer at age 38 caused significant distress. The journey from a persistent nipple fissure to a diagnosis of MPD was fraught with anxiety. Learning that the imaging showed no deep mass provided temporary relief, but the recommendation for sentinel lymph node biopsy based on other high-risk features was a critical turning point. The discovery of lymph node metastases was devastating, yet the clarity it provided was crucial. It meant my treatment could be tailored accurately to the true extent of my disease.

The subsequent chemotherapy, targeted therapy, and radiotherapy were challenging, but were made manageable by the proactive support from my healthcare team. The psychological counseling I received was invaluable in alleviating my fear and anxiety.

Now, with treatment completed and follow-ups showing no recurrence, I have returned to a normal life. I am grateful for the thorough staging and precise treatment, which I believe was pivotal for my current well-being. This experience underscores that beyond advanced medicine, clear communication and compassionate care are fundamental to healing.

The patient has reported considerable satisfaction with the treatment results. Following the initial diagnosis, she experienced significant psychological distress, including anxiety, tension, memory decline, and sleep disturbances. Through repeated counseling sessions with our hospital’s psychological intervention team and detailed explanations from our healthcare staff, her fear and anxiety related to the disease were gradually alleviated. With consecutive follow-up visits showing no recurrence, she has successfully reintegrated into a normal life trajectory. Currently, the patient reports no specific discomfort, with well-healed surgical wounds. She wears an external prosthesis and expresses satisfaction with the postsurgical cosmetic outcome.

The timeline of key symptoms, surgeries, pathology, treatments, and follow-up events is summarized in **Table 1**.

**Table 1:** Timeline of Symptoms, Investigations, Surgeries, Pathology, Treatments, and Follow-up.

Date	Category	Event	Key findings
Early 2022	Symptom	Symptom Onset	Recurrent left nipple fissure, accompanied by mild discomfort.
Dec 2024	Symptom	Disease Progression	Eczematous changes (erythema, papules, exudation) over the left nipple-areola complex.
Jan 2025	Pathology	Initial Diagnosis: Nipple skin biopsy	Local hospital pathology confirmed MPD. IHC: ER (–), PR(-), HER2(3+), Ki-67 75%.
Feb 2025	Investigations	Preoperative Imaging Evaluation	Ultrasound, mammography, and MRI showed no definite deep mass, only areolar skin thickening and nodular enhancement posterior to the areola.
Feb 2025	Surgery	First Surgery: Left Breast Partial Extended Resection + SLNB	Intraoperative frozen section of 4 sentinel LNs revealed 2 with macrometastases and 2 with micrometastases.
Feb 2025	Surgery	Additional Axillary Lymph Node Dissection	Intraoperative Decision: According to guidelines, left ALND was performed immediately based on positive SLNB findings.
Feb 2025	Pathology	Final Pathology Report of First Surgery	Breast: MPD with multifocal microinvasive carcinoma (pT1mic). LNs: Macrometastases in axillary (6/8) and subclavian (2/2) LNs.
Feb 2025	Investigations	Postoperative PET-CT	Revealed lymph nodes with increased uptake in the left supraclavicular fossa, suggestive of metastasis.
Feb 2025	Surgery	Second Surgery : Left Total Mastectomy + Left Cervical Lymph Node Dissection	Aimed at complete resection. Pathology confirmed macrometastasis in 1 of 6 left supraclavicular LNs. Final pathological stage: pT1mic pN3c cM0 (Stage IIIC)
Mar 2025 to Jun 2025	Treatment	Postoperative Systemic Therapy	Based on MDT discussion, HER2-positive subtype, and pN3c stage, the patient received the TCbHP regimen with radiotherapy.
July 2025	Investigations	Follow-up PET-CT Scan	No recurrence was observed
Sep 2025 to Dec 2025	Treatment	Consolidation Therapy	Considering high nodal tumor burden, oral Capecitabine was administered as consolidation therapy.
Dec 2025	Follow-up	Follow-up Ultrasound	No evidence of recurrence; patient asymptomatic.

MPD, Mammary Paget’s Disease; IHC, Immunohistochemistry; SLNB, Sentinel Lymph Node Biopsy; ALND, Axillary Lymph Node Dissection; LN, Lymph Node; LND, Lymph Node Dissection; PET-CT, Positron Emission Tomography-Computed Tomography; MDT, Multidisciplinary Team; TCbHP, docetaxel, carboplatin, trastuzumab, and pertuzumab.

## 3 Discussion

This case reports a rare presentation of MPD: no definite breast mass was detected on imaging, but postoperative pathology confirmed concurrent multifocal microinvasive carcinoma and extensive lymph node metastasis (pN3c) in the axillary and clavicular regions. The findings from this single case challenge the assumptions underlying existing clinical guidelines and highlight the potential risks of understaging. However, the generalizability of these findings needs to be confirmed in larger studies.

### 3.1 Limitations of preoperative evaluation for occult lesions associated with MPD

Current guidelines, including those from the National Comprehensive Cancer Network (NCCN) and the Chinese Society of Clinical Oncology (CSCO), provide a rationale for omitting SLNB in selected patients with MPD. This rationale is predicated on two key assumptions: first, that a comprehensive preoperative evaluation (encompassing physical examination, imaging, and full-thickness biopsy) can reliably identify the presence of an underlying invasive carcinoma; and second, that the risk of lymph node metastasis in “pure” MPD or DCIS is sufficiently low to justify foregoing surgical staging (19, 20). The NCCN guidelines state that axillary staging may be omitted for patients undergoing breast-conserving surgery for MPD if there is no radiological or clinical evidence of invasion (19). Similarly, the CSCO guidelines reference this principle, indicating that some experts support omitting SLNB when imaging excludes DCIS or invasive cancer (2, 20).

The present case exposes critical gaps in this rationale. Firstly, the “comprehensive” preoperative evaluation failed to detect the multifocal microinvasive carcinoma, which was only identified upon exhaustive postoperative histological sectioning. This underscores a well-documented limitation of imaging. Invasive foci may elude detection on MRI due to their minute size or diffuse growth pattern (9). Furthermore, DCIS lesions often present as non-mass-like, clumped enhancements with segmental or linear distribution and low peak enhancement, making them particularly challenging to discern, especially against a background of parenchymal enhancement (8). The false-negative imaging in this case is not an anomaly but rather a reflection of the inherent resolution limit of current technology in detecting microscopic invasion.

### 3.2 Microinvasive carcinoma and extensive lymph node metastasis: a challenge to traditional cognition

The most striking aspect of this case is the profound discrepancy between the minimal primary tumor burden and the advanced nodal disease. This finding challenges the conventional paradigm directly linking tumor size to metastatic potential (21, 22). Several non-mutually exclusive mechanisms may explain this phenomenon. First, the anatomical location of the primary disease in the central/subareolar region is critical (12, 13). This area contains a dense lymphatic network, which may facilitate early tumor cell dissemination even from minute invasive foci (23, 24). Second, the aggressive HER2-positive subtype is strongly associated with a high propensity for lymphatic invasion and metastasis, largely independent of tumor size (10). The cancer cells may exhibit an innate metastatic phenotype, enabling distant seeding at a very early stage. Third, the identified sub-1 mm foci might represent only the visible portion of a more substantial invasive process that was not fully captured during histological sampling. The absence of LVI, while noted, does not definitively preclude the possibility of intermittent tumor shedding into lymphatic channels (11). This case underscores that in MPD, particularly with high-risk features such as a central location and HER2 positivity, the pathological T-stage can be an unreliable predictor of nodal status. Sole reliance on T-stage for surgical decision-making risks significant understaging.

### 3.3 Contrast with guideline recommendations and clinical implications

This case presents a finding that appears to challenge the current guideline recommendations, which permit the omission of SLNB in selected patients with MPD and no radiologic evidence of invasion (19, 20). The NCCN and CSCO guidelines are predicated on the general assumptions that preoperative evaluation can reliably exclude invasive carcinoma and that the nodal metastasis risk in pure MPD is low. The experience from our case, however, may highlight a potential scenario where these assumptions do not hold, underscoring how accurate nodal staging can profoundly impact perceived prognosis and therapeutic decisions.

In this patient, the initial presentation—MPD with no definitive mass on MRI—might have, if guidelines were strictly followed, led to understaging. The decision to perform SLNB, driven by aggregate high-risk features, proved critical. It revealed axillary nodal metastases, which upgraded the pathological stage and altered the treatment paradigm. This diagnostic cascade ultimately led to the final stage of pT1mic pN3c. One might speculate that without SLNB, the patient could have been understaged and potentially under-treated.

The intensified, multimodal adjuvant regimen administered to this patient stands in stark contrast to the treatment that would have been indicated for a pure MPD without invasive carcinoma or lymph node involvement. This disparity illustrates how the pathologic findings from SLNB and subsequent surgeries directly dictated a therapeutic escalation that was critical for addressing the patient’s true disease burden. Specifically:

Systemic Therapy (TCbHP regimen): The diagnosis of stage pN3c, HER2-positive disease mandated an anti-HER2 targeted therapy combined with chemotherapy (19). This TCbHP regimen would not be indicated for a patient with pure, node-negative MPD or DCIS, highlighting how the discovery of occult nodal metastasis altered the systemic treatment approach to target the aggressive biology of the invasive component.

Comprehensive Radiotherapy: The extensive lymph node metastasis (involving axillary, subclavian, and supraclavicular regions) necessitated adjuvant radiotherapy targeting not only the residual breast tissue (post-mastectomy) but also the supraclavicular and infraclavicular nodal basins. This extensive field is justified by the pN3c staging (17). For pure MPD, radiotherapy would typically be limited to the breast or chest wall (25).

Consolidation Therapy (Capecitabine): The decision to employ capecitabine consolidation was based on the high nodal tumor burden (pN3c), a strategy supported by evidence for high-risk breast cancer (18). This represents an additional layer of treatment intensity directly attributable to the accurate staging made possible by SLNB.

In conclusion, this case demonstrates that the omission of SLNB would likely have resulted in treatment limited to local therapy for the nipple-areola complex, grossly inadequate for the patient’s actual pT1mic pN3c stage. The SLNB procedure was, therefore, not merely a diagnostic tool but a critical intervention that unlocked the precise, stage-appropriate, and potentially curative multimodal therapy that the patient ultimately received.

## 4 Conclusion

In summary, this single case report suggests that MPD can be associated with microinvasive carcinoma possessing high metastatic potential, even with negative preoperative imaging. Our findings highlight a potential limitation in the guideline recommendation of omitting SLNB based solely on preoperative evaluation and may help generate the hypothesis that SLNB retains crucial staging value in this specific context. We propose that clinical decision-making should incorporate​ an individualized risk assessment, and our observations would benefit from further validation to refine future guidelines.

### 4.1 Resource identification initiative

No biological resources (e.g., antibodies, cell lines) requiring catalog numbers or RRIDs were used in this study.

### 4.2 Life science identifiers

This study does not involve ZOOBANK-registered names or nomenclatural acts; therefore, no relevant LSIDs need to be indicated.

## Data Availability

The datasets presented in this article are not readily available because This study is a clinical case report of Paget’s disease of the breast. No datasets such as genetic sequencing or omics analysis were generated, so there are no applicable dataset restrictions. Meanwhile, in accordance with medical ethical norms, any information related to patients must not be used for identification or dissemination for non-research purposes. Requests to access the datasets should be directed to the corresponding author.
