# Supplementary material for: Case Report: Mammary Paget’s disease with multifocal microinvasive carcinoma and extensive lymph node metastasis: therapeutic challenges and insights from a case of stage pT1mic pN3c cM0
Source: Front Oncol. 2026 Jan 21;15:1727016. doi: 10.3389/fonc.2025.1727016 (PMC12867909; doi:10.3389/fonc.2025.1727016)
Supplement: Supplementary file 1 [file DataSheet1.pdf]

Date	Category
**Early 2022**	Symptom
**Dec 2024**	Symptom
**Jan 2025**	Pathology
**Feb 2025**	Investigations
**Feb 2025**	Surgery
**Feb 2025**	Surgery
**Feb 2025**	Pathology
**Feb 2025**	Investigations
**Feb 2025**	Surgery
**Mar 2025 to Jun 2025**	Treatment
**Sep 2025 to Dec 2025**	Treatment
**Jun 2025 to Dec 2025**	Follow-up

Abbreviations: MPD, Mammary Paget's Disease; IHC, Immu

Event
Symptom Onset
Disease Progression
Initial Diagnosis: Nipple skin biopsy
Preoperative Imaging Evaluation
First Surgery: Left Breast Partial Extended Resection + SLNB
Additional Axillary Lymph Node Dissection
Final Pathology Report of First Surgery
Postoperative PET-CT
Second Surgery：Left Total Mastectomy + Left Cervical Lymph Node Dissection
Postoperative Systemic Therapy
Consolidation Therapy
Follow-up

nohistochemistry; SLNB, Sentinel Lymph Node Biopsy; ALND, Axillary Lymph Node Dissec

Key Findings
Recurrent left nipple fissure, accompanied by mild discomfort.
Eczematous changes (erythema, papules, exudation) over the left nipple-areola complex.
Local hospital pathology confirmed MPD. IHC: ER(-), PR(-), HER2(3+), Ki-67 75%
graphy, and MRI showed no definite deep mass, only areolar skin thickening and nodular enhancement p
traoperative frozen section of 4 sentinel LNs revealed 2 with macrometastases and 2 with micrometastase
erative Decision: According to guidelines, left ALND was performed immediately based on positive SLNB f
D with multifocal microinvasive carcinoma (pT1mic). LNs: Macrometastases in axillary (6/8) and subclaviar
Revealed lymph nodes with increased uptake in the left supraclavicular fossa, suggestive of metastasis.
ion. Pathology confirmed macrometastasis in 1 of 6 left supraclavicular LNs.Final pathological stage: pT1
MDT discussion, HER2-positive subtype, and pN3c stage, the patient received the TCbHP regimen with rac
Considering high nodal tumor burden, oral Capecitabine was administered as consolidation therapy.
No recurrence was observed, and the patient recovered well.

ction; LN, Lymph Node; LND, Lymph Node Dissection; PET-CT, Positron Emission Tomography-Computed
